# Investigating sex differences, cognitive effort, strategy, and performance on a computerised version of the mental rotations test via eye tracking

**DOI:** 10.1038/s41598-019-56041-6

**Published:** 2019-12-19

**Authors:** Adam J. Toth, Mark J. Campbell

**Affiliations:** 1 Department of Physical Education & Sport Sciences, University of Limerick, Castletroy, Limerick Ireland; 2 Lero, Irish Software Research Centre, University of Limerick, Castletroy, Limerick Ireland

**Keywords:** Human behaviour, Attention

## Abstract

Mental rotation tests (MRTs) have previously shown one of the most prominent sex differences in cognitive psychology, marked by a large male performance advantage. However, debate continues over the reasons for these sex differences. Previously, we used pupillometry to demonstrate sex differences in the cognitive effort invoked during the original MRT. Here, we evaluated the magnitude of sex differences during performance on a computerized version of the Vandenberg and Kuse MRT. Secondly, we examined whether fixation metrics could illuminate strategy use by participants. Finally, we used pupillometry to investigate whether cognitive effort differed between sexes and trials of different difficulty. While our results demonstrate no performance differences between sexes on the computerized MRT, fixation patterns provided evidence that gaze strategy was associated with performance on different parts of the test. Moreover, we show the cognitive demand of the V&K MRT, evidenced by large task dependent increases in participants’ pupil diameters.

## Introduction

Spatial cognition is critical for our ability to successfully orient ourselves^1^ and navigate our environment^2^. It has been linked to success on IQ tests^3^ and with performance in STEM (Science, Technology, Engineering and Mathematics) subjects^4^. As a result, understanding how to improve and evaluate spatial cognitive abilities remains topical in psychological science and cognitive neuroscience. While a number of methods exist to evaluate different aspects of spatial cognition, mental rotation tests garner the most widespread attention among researchers. Mental rotation ability has been increasingly studied since the early 1970s and superior performance on mental rotation tests has been associated with spatial navigation performance^5^ higher intelligence^6^ and better motor action performance^7^. Moreover, tests of mental rotation have previously shown one of the most prominent sex differences in cognitive psychology, marked by a large male performance advantage^8,9^.

**Figure 1:**
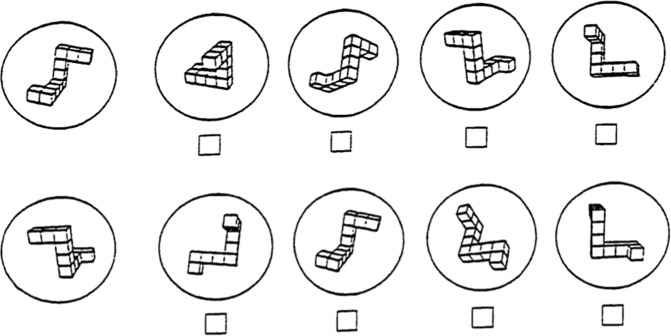
Two sample trials from the computerized MRT^11^ used in the current study. In each trial a standard image (*left*) and 4 test images were presented, 2 of which (*1 and 3*) were identical rotated versions of the standard, and two of which were mirror (*top*) or structural (*bottom*) foils.

Shepard and Metzler^10^ developed the original mental rotations test (MRT), where participants compared a pair of 3D cube images and judged whether the test image on the right was the same or a mirror rotated version of the standard image on the left. By including test stimuli that varied in their degree of rotation from the standard image, the original MRT shows that reaction times increase and accuracy degrades as the degree of rotation increases between the two stimuli. Following this, another version of the MRT was developed by Vandenburg and Kuse^11^. In this paper and pencil test, participants are presented with a standard cube image and 4 test cube images (see Fig. 1 for Vandenberg and Kuse examples) and must judge which 2 of the 4 images are identical rotated versions of the standard image. Typically, 20–24 trials are included on this test, with half of the trials including mirror foils of the standard image and half of the images including structural foils. Structural foils are cube images where one of the ends of the cube figure are oriented differently compared to the standard image and it is hypothesized that the ability for a participant to exclude these foils as being the same as the standard is facilitated relative to mirror foils. While the original MRT is typically performed on a computer, where precise reaction times (RT’s) can be recorded for repeats of a given angle, the V&K MRT test is typically performed on paper and participants are given either 3 minutes for 2 sections of 10–12 trials^8,12^ or 10–15 minutes to complete all trials on the test^13,14^. More recently, researchers have adapted the V&K MRT stimuli and presented computerized versions of this test^13,15^, as these versions have the advantage of being more accurate and easily administered to large numbers of people^16^.

Despite the fact that the V&K MRT has previously shown one of the largest sex differences in cognitive psychology, debate has continued over the nature and even existence of this sex difference. Generally, the V&K MRT shows the largest male advantage while alternative tests, including the original MRT, show a more modest male advantage^17^. However, as more research has been devoted to studying MRT performance, it has been noted that when MRT administration criteria are manipulated, the sex difference diminishes. For example, when participants are not time constrained on the V&K and original MRTs, the sex difference is significantly diminished^18,19^ and even abolished^20,21^ respectively. It has also recently been shown that when female confidence is high regarding their perceived MR ability, or when the abstract cube test stimuli are swapped for hands test stimuli, their performance does not differ from their male counterparts^22,23^. As a result, a body of literature has surfaced suggesting the purported sex difference can be attributed to a number of factors beyond mental rotation skill, including genetic^17^, evolutionary^24^, social^25^, cognitive^23^ and performance factors^14^.

One important performance factor that has been identified is the strategy adopted to complete the MRT^26,27^. For the V&K MRT, it has been argued that females employ a more cautious strategy compared to men^28^, particularly when deciding whether mirror foil images match the standard image^29^. Alternatively, it has been argued that males tend to rely on a ‘leaping’ strategy whereby they progress to the next trial as soon as they have identified matching images^30^. Although previous work to date has relied on surveys/questionnaires following completion of the MRT to gauge response strategies^9^, eye tracking has been more recently utilised in an attempt to objectively quantify the success of different strategies during completion of the original MRT. However, findings remain inconclusive as the ratio of visual fixations on test versus standard images on the original MRT has been used to suggest paradoxically that a piecemeal strategy (ratio of fixations within an image: fixations between images > 1) is both more^31^ and less^32^ successful compared to a holistic strategy (ratio = 1). While some work to date has investigated gaze strategies on the original MRT, no work to date has explored the gaze strategies deployed by either sex when completing the V&K MRT, where the number of possible options, and thus fixation locations, on any given trial is doubled. Also, when using eye tracking during the completion of a task with multiple options, it has been demonstrated that participants often choose the option they paid more attention to^33^. Thus, examining fixation durations and fixation counts on the V&K MRT may yield insights into the adoption of a new strategy or a previously described strategy among participants when specifically performing the V&K test of mental rotation.

A further benefit to the use of eye tracking technology during cognitive tasks is the ability to record pupillometric data. Changes in pupil diameter under constant luminance have long been shown to indicate the cognitive effort invested during completion of a given task^34^. Specifically, as the difficulty of a task increases, requiring more cognitive effort or resources, concurrent increases in pupil diameter are observed. Others have described changes in pupil diameter as an index of how intensely the processing system is operating^35,36^. Pupil dilation with increasing task difficulty has been shown during the recall of longer number sequences^37,38^, the multiplication of larger numbers^39^, the recall of sentences that differed in syntax complexity and sentence length^40^ and even between congruent and incongruent conditions on the color word stroop task^41^. Task related fluctuations in pupil diameter are correlated with activity in the dorsal attention network (DAN), dorsolateral prefrontal cortex (DLPFC), thalamus, superior colliculus (SC) and the Locus Coeruleus, which exerts influence on frontal lobe activity^42^. As a result, it is not surprising that researchers have found a relationship between pupil dilation and executive functioning and working memory. Recently, pupillometry measures have been used to highlight that during the original MRT, females display larger increases in pupil diameter and thus, exert significantly more cognitive resources toward the mental rotation of abstract cube images compared to males in order to achieve a similar performance^23^. However, no work to date has used pupillometry to evaluate how much cognitive effort is deployed by males and females on the different trial types of the V&K MRT.

In the current study we first aimed to evaluate the magnitude of the sex difference during performance on a computerized version of the V&K MRT in a large sample of young healthy adults. Secondly, we looked to determine whether fixation duration and fixation count metrics could provide an objective indication of the strategy used by participants as they completed the test. Finally, we aimed to investigate differences in the cognitive effort deployed by males and females on trials of different difficulty over the test. We first hypothesized, in line with previous work, that males would perform superiorly compared to females on both trial types (mirror and structural foils) of the V&K MRT, as indicated by higher scores and shorter test completion times. Secondly, we hypothesized that participants would find trials with mirror foils more difficult than trials with structural foils. Thirdly, we hypothesized that when individuals spent more time fixating or focusing their attention upon images that are the same as the standard image (correct responses), they would tend to score higher. Finally, based on previous work in our lab, we hypothesized that females would exert more cognitive effort, indicated by larger increases in pupil diameter, than males when performing the V&K MRT.

## Results

### MRT performance metrics

**Figure 2:**
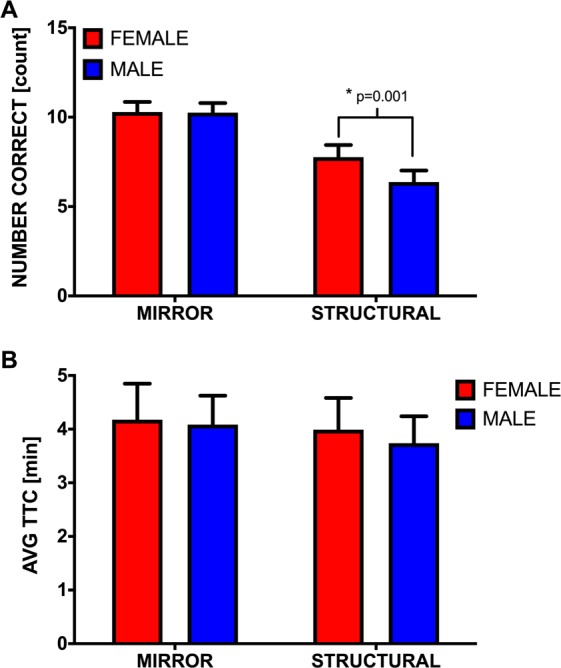
Mean number of correct responses (**A**) and average Times to Completion (TTC) (**B**) on Mirror and Structural foil trials. Data are expressed as means ± 95% CI.

All participants could complete the computerized MRT within the maximum allowed time of 15 minutes. A description of all measures can be found in the Methods section of the manuscript. A Pearson’s correlation between score and Reaction Time (RT) for data from all individual trials completed by all participants were not significant (r = 0.018, n = 1400, p = 0.517). A mixed ANOVA demonstrated a significant Sex*Foil Type interaction (F_(1,68)_ = 8.237, p = 0.006, η^2^ = 0.111) for MRT score. Post hoc comparisons revealed that both male (p < 0.001, η^2^ = 0.584) and female (p < 0.001, η^2^ = 0.392) participants scored significantly higher on Mirror compared to Structural foil trials and that female participants scored significantly higher than males on the Structural (p = 0.001, η^2^ = 0.168), but not Mirror (p = 0.911, η^2^ = 0.000), foil trials (see Fig. 2A). A significant main effect of *Foil Type* was found for Time to Completion (TTC) data (F_(1,68)_ = 9.256, p = 0.003, η^2^ = 0.123) with participants taking approximately 17.283 s longer on average to complete mirror compared to structural foil trials on the MRT (see Fig. 2B). No main effect was found for *Sex* (F_(1,68)_ = 0.071, p = 0.791, η^2^ = 0.001) nor was the Sex*Foil Type interaction significant (F_(1,68)_ = 1.335, p = 0.252, η^2^ = 0.020).

### Fixation data

A mixed ANOVA showed no significant main effects [*sex*; (F_(1,68)_ = 0.011, p = 0.918, η^2^ = 0.000), *foil*; (F_(1,68)_ = 0.945, p = 0.334, η^2^ = 0.014)] or sex*foil interaction (F_(1,68)_ = 2.955, p = 0.090, η^2^ = 0.043) for Total Fixation Duration (TFD) on Standard images. However, a significant sex*foil type interaction was found for Fixation Count (FC) on Standard images (F_(1,68)_ = 5.787, p = 0.019, η^2^ = 0.081). Post hoc comparisons showed that only male participants directed a larger number of fixations toward standard images on mirror foil compared to structural foil trials (p = 0.001, η^2^ = 0.148).

**Figure 3:**
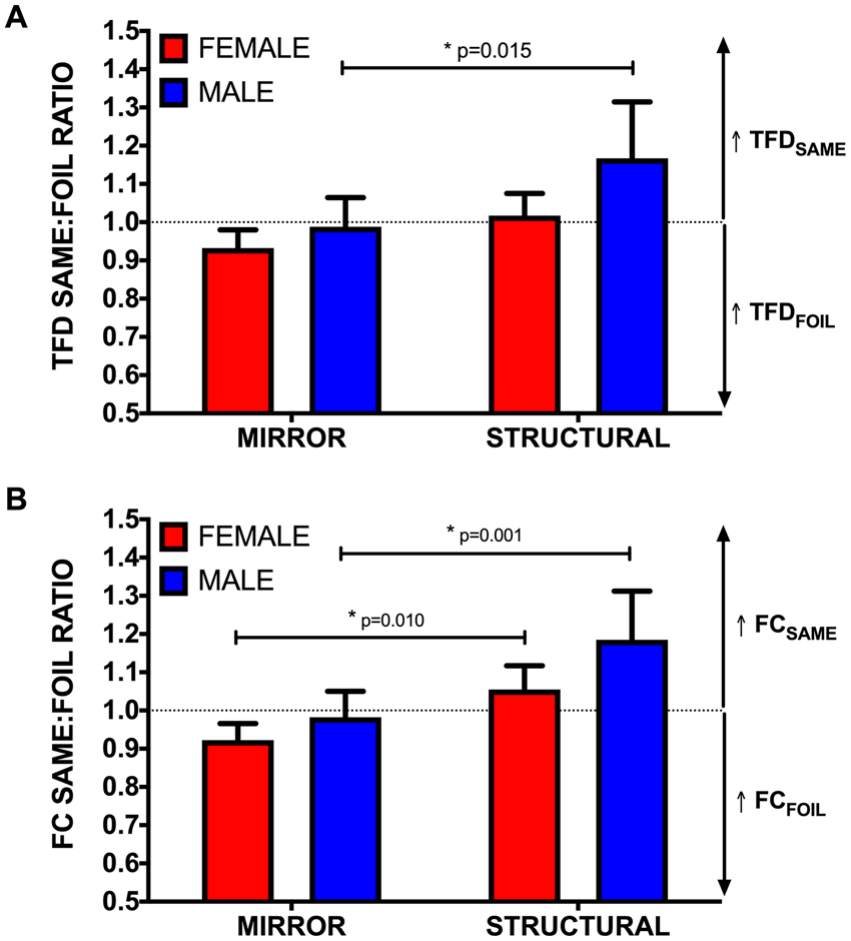
The ratio of total fixation duration (TFD) (**A**) and number of fixations (fixation count: FC) (**B**) on same images relative to foil images. The dotted line represents equal TFDs or FCs on same and foil images. Ratios above and below 1 represent longer TFDs or more FCs on same and foil images respectively. Error bars indicate ± 95% CI and ^*^indicates significance at p < 0.05.

When analysing TFD same:foil ratios, a significant main effect was found for both *sex* (F_(1,68)_ = 4.441, p = 0.039, η^2^ = 0.063) and *foil type* (F_(1,68)_ = 8.023, p = 0.006, η^2^ = 0.108). Because only comparisons between sexes within a given foil trial type and between foil types within a given sex were of interest (i.e. comparisons between male performance on structural foil trials and female performance on mirror foil trials were irrelevant) we conducted planned comparisons that revealed that males specifically had significantly longer TFDs on ‘same’ images that were rotated versions of the standard when responding to structural compared to mirror foil trials (p = 0.015, η^2^ = 0.087) (see Fig. 3A). A mixed ANOVA performed on FC data similarly revealed main effects for both *sex* (F_(1,68)_ = 4.668, p = 0.034, η^2^ = 0.066) and *foil type* (F_(1,68)_ = 19.118, p < 0.001, η^2^ = 0.225), with planned comparisons showing that both males (p = 0.001, η^2^ = 0.156) and females (p = 0.010, η^2^ = 0.096) directed a larger number of fixations toward images that were rotated versions of the standard for structural compared to mirror foil trials (see Fig. 3B).

**Figure 4:**
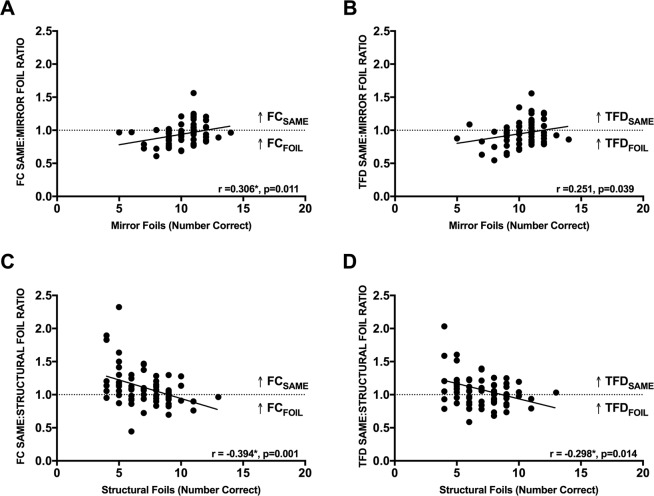
(**A**,**B**) Scatter plots between the number of correct responses on mirror foil trials and the ratio of fixation count (FC) and total fixation duration (TFD) between same and mirror foil images. (**C**,**D**) Scatter plots between the number of correct responses on structural foil trials and the ratio of fixation count (FC) and total fixation duration (TFD) between same and structural foil images. The dotted line represents the threshold between having a higher fixation count or longer total fixation duration on same or foil images. *Represents significant correlations (r) at a Sidak adjusted p < 0.025.

Pearson’s correlations between same:mirror ratios and scores on mirror foil trials revealed that for both TFD and FC, the more fixations and longer TFDs on images that were rotated images of the standard, the better participants scored on mirror foil trials (FC: r = 0.306, n = 70, p = 0.011; TFD: r = 0.251, n = 70, p = 0.039) (see Fig. 4A,B). However, correlations between same:structural ratios and scores on structural foil trials revealed that for both TFD and FC, the more fixations and longer TFDs on images that were rotated images of the standard, the worse participants scored on structural foil trials (FC: r = −0.394, n = 70, p = 0.001; TFD: r = −0.298, n = 70, p = 0.014) (see Fig. 4C,D).

### MPD changes

**Figure 5:**
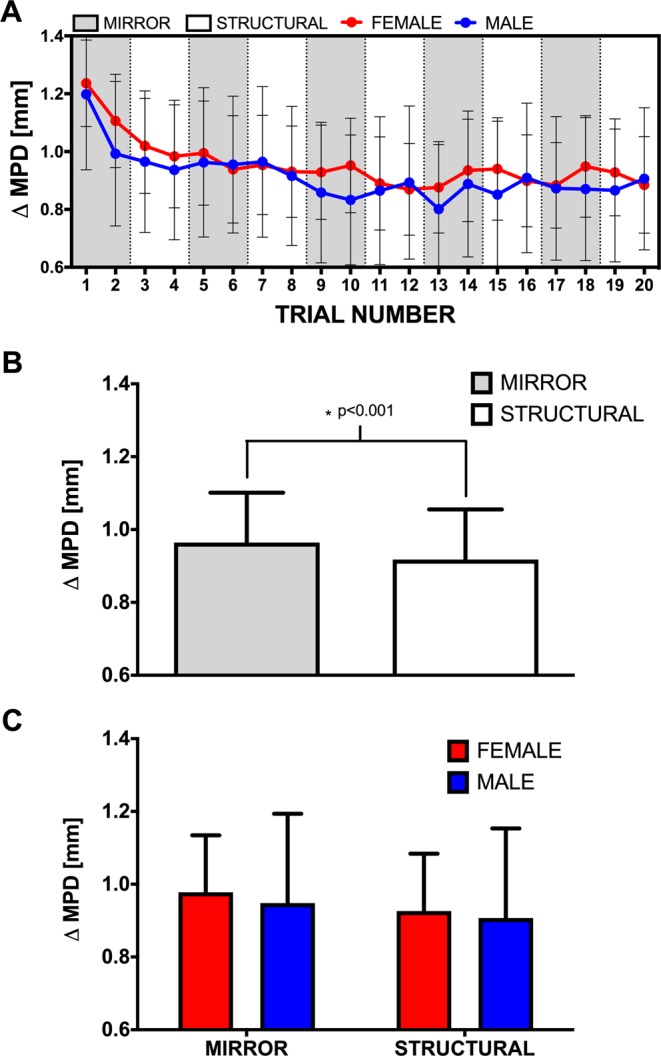
(**A**) Max change of pupil diameter relative to baseline (**Δ** MPD) across all 20 trials of the MRT. White and grey sections highlight trials with structural and mirror foils respectively. (**B**) Average **Δ** MPD for all participants and for males and females (**C**) across mirror and structural trials. Error bars indicate ± 95% CI and ^*^indicates significant differences at p < 0.05.

A mixed ANOVA revealed a significant main effect for *foil type* (F_(1,68)_ = 33.145, p < 0.001, η^2^ = 0.334), with participants displaying larger changes in Maximum Pupil Dilation (MPD) for mirror foil trials compared to structural foil trials. The effect of *sex* (F_(1,68)_ = 0.282, p = 0.597, η^2^ = 0.004) and the sex*foil type interaction (F_(1,68)_ = 0.005, p = 0.943, η^2^ = 0.000) were not significant (Fig. 5).

## Discussion

This study set out to evaluate performance on a computerized version of the V&K MRT and investigate, using novel pupillometry and gaze measures, the purported sex difference in performance on this task^8,9,20^. Firstly, we found no significant performance difference between males and females on mirror foil trials and, in fact, we instead observed that males scored more poorly compared to females on structural foil trials. Both males and females also took similar amounts of time to complete the test and overall, participants scored better on mirror compared to structural foil trials. Second, we showed that all participants displayed large increases in pupil diameters during completion of the MRT, evidence of the cognitive demand of the task. While the amount of cognitive effort used to complete the test was not different between males and females, larger dilations were observed for mirror compared to structural foil trials. This provides evidence that more cognitive effort was contributed toward the completion of mirror foil trials compared to structural foils. Third, we discovered an association between fixation patterns and performance among all participants. Specifically, when participants spent relatively more time fixated upon same foils (correct options), they performed better on mirror foil trials but worse on structural foil trials. These findings are discussed within the context of the existing literature.

### Performance sex differences

The similar performance of males and females on the MRT in this study contradicts previous assertions that mental rotation ability is marked by large sex differences, favouring male performance. Previous literature has debated the nature of sex differences in mental rotation and despite a plethora of evidence to suggest that males tend to out-perform females on the V&K MRT, a recent growing body of evidence suggests that performance factors may be involved and may moderate the size of the purported sex difference. For example, Voyer^19^ demonstrated that the sex difference is larger when time is constrained. Many tests provide 3 minutes per set of 10 trials^8,9^. However, when more time is allotted, the sex difference is weakened or abolished. In accordance with a computerized MRT used by Strong^13^, we gave participants 15 minutes to complete the test, well above the prescribed 6 minutes, and despite the fact no participants ran out of time (average completion time of 8.069 minutes), many may have under previous time restriction criteria. The removal of the standard time limit of 10 min to complete the MRT has led some to conclude that there is no sex difference^18,43^, while others show a reduction in magnitude^28^. Based on the previous literature, the loosely restricted completion time used in this study may have provided more time for especially female participants to perform better than they may have done with a stricter time constraint.

Additionally, the difference we observe between males and females for structural foil trials may have resulted from the method of scoring we adopted from Strong^13^. Many studies^11,44^ score the V&K MRT by only awarding points when both answers for a given trial are correct, or when one answer is correct without a further attempt (i.e. no incorrect answers). Our program required participants to input two answers for every trial before being able to move on to the next trial, making guessing on at least one answer for any trial a very real possibility. To reduce or eliminate the likelihood of guessing^11^, we also scored the test by awarding a point for only trials with two correct answers (Table 1). When scoring in this way, no difference was found between males and females for trials of either foil type.

**Table 1:** Means ± 95% CI for all dependent variables for Male, female and all participants on Mirror foil, structural foil, and all trials on the V&K MRT.

Sex	Foil Type	Score (*All Correct*)	Score (*2 Correct*)	TTC (min)	Δ MPD (mm)	FC (Standard)	FC (*SAME:FOIL*)	TFD (Standard)	TFD (*SAME:FOIL*)
MALE	MIRROR	10.333 ± 0.510	2.033 ± 0.206	4.181 ± 0.404	0.905 ± 0.235	219.133 ± 34.483	0.980 ± 0.069	64.978 ± 11.505	0.982 ± 0.078
	STRUCTURAL	6.133 ± 0.521	0.467 ± 0.124	3.765 ± 1.007	0.854 ± 0.233	194.067 ± 32.373	1.176 ± 0.129	59.688 ± 11.462	1.147 ± 0.149
	TOTAL	16.467 ± 0.762	2.500 ± 0.270	7.946 ± 1.007	0.879 ± 0.233	413.200 ± 65.676	—	124.667 ± 22.401	—
FEMALE	MIRROR	10.289 ± 0.549	2.132 ± 0.189	4.176 ± 0.649	0.978 ± 0.151	205.763 ± 34.680	0.923 ± 0.041	62.458 ± 11.783	0.932 ± 0.047
	STRUCTURAL	7.763 ± 0.658	0.842 ± 0.144	3.989 ± 0.572	0.926 ± 0.153	204.526 ± 35.725	1.054 ± 0.061	63.926 ± 11.742	1.017 ± 0.057
	TOTAL	18.053 ± 0.858	2.974 ± 0.243	8.166 ± 1.194	0.952 ± 0.151	410.289 ± 69.000	—	126.384 ± 22.861	—
ALL	MIRROR	10.309 ± 0.376	2.088 ± 0.139	4.178 ± 0.429	0.946 ± 0.133	211.662 ± 24.510	0.948 ± 0.039	63.570 ± 8.259	0.954 ± 0.043
	STRUCTURAL	7.044 ± 0.472	0.677 ± 0.099	3.890 ± 0.390	0.895 ± 0.133	199.912 ± 24.404	1.108 ± 0.067	62.056 ± 8.240	1.074 ± 0.072
	TOTAL	17.353 ± 0.611	2.765 ± 0.182	8.069 ± 0.796	0.920 ± 0.133	411.574 ± 47.885	—	125.627 ± 16.037	—

*All Correct* scores were calculated by summing all individual correct responses. *2 Correct* scores were calculated by summing all points, where a point is awarded only for trials where both ‘same’ images were identified.

Another factor identified within the MRT literature that is argued to potentially affect performance between the sexes is that of a perceived stereotype regarding superior spatial cognition in males^22^. Previous work has shown that prior expectation about how one might be expected to perform on the MRT can influence that individual’s score^45^. Moreover, one study has shown that when reducing the motivational barriers via training, the mental rotation sex difference can be eliminated and, in some cases, reversed^46^. Participants in this study were not informed or aware of any existing sex difference prior to participation in the study and the lack of sex differences in score could reflect motivational differences between the groups that either led females to perform better than they may have otherwise or males to perform worse. We look to future research to investigate the impact of performance expectation and motivation on tasks of spatial cognition, including mental rotation tests.

### Gaze strategy

Our eye tracking data highlight that performance on the MRT was significantly associated with where participants allocated their attention. Specifically, we found that on mirror trials, those who directed their visual attention toward the mirror foils more often or for longer compared to same foils, performed more poorly on these trials. Alternatively, participants who directed their visual attention on structural foils more often or for longer performed better on the structural trials. Previous literature has investigated some of the different strategies employed by those completing mental rotation tasks. Some of these strategies include mentally changing one’s viewing perspective versus mentally rotating the object^47^, an analytic (feature based, orientation independent) versus global shape or motor simulation-based strategy^48,49^, and a leaping strategy versus a more cautious approach^28^. The gaze strategy or behaviour displayed by participants in this study may be linked to the previously reported ‘leaping’ strategy^48^. In this way, those who attended more toward mirror foils might have been more prone to mistakes with a leaping strategy. This is because without a cautious approach, participants may have been more likely fooled into thinking they had the correct answer when gazing upon the mirror foils more compared to the same foils. Alternatively, those who attended more toward structural foils would likely have an advantage if adopting a leaping strategy as they may be better able to rule out the obvious ‘different’ test images compared to identifying the ‘same’ test images. However, this strategy was thought to be employed predominantly by male participants^28^. Here, while we do find that males had more fixations and longer fixation durations on same foils (Fig. 3), which may partly explain their decreased performance on structural foil trials (Fig. 2), we found overall that both male and female participants benefited from the observed gaze strategies (Fig. 4) and given that TTCs did not differ between the sexes, it is not likely that different leaping and cautious strategies were used between the sexes. Although the specific strategy participants adopted during completion of the test was not recorded anecdotally, future work might look to investigate the association between reported strategies on the MRT using post-test questionnaires/surveys and in test fixation patterns. However, we report for the first time evidence that fixation patterns indicating differences in attention allocation on mirror and structural foils might serve as a biological marker confirming the adoption of a leaping strategy when completing the V&K MRT.

### Pupillometry

When examining the pupillometry data, we found that all participants exhibited increases in pupil dilation relative to their own baselines during completion of the MRT. We also found that the magnitude of dilation was significantly greater for mirror, compared to structural trials. This task dependent change in pupil dilation has been shown to indicate differences in the cognitive effort allocated over the course of performing a number of different tasks^23,34,41^. However, despite recent evidence suggesting females exert more cognitive effort toward completing the original MRT task^23^ we did not find any difference in MPDs between males and females for trials of either foil type on the V&K MRT. This suggests that both males and females are contributing similar amounts of cognitive effort toward completing this specific MRT task.

It is also noted that pupil dilations in this study are relatively high compared with those found in participants who completed the original MRT (0.4–0.7 mm diameter increases)^23^ or during the completion of other cognitive tasks. Other tasks, such as multiplication, digit transformation, number recall, and target tracking tasks see max pupil dilations during difficult trials of approximately 0.5mm^39^, 0.7mm^50^, 0.5mm^37^, and 0.4mm^42^ respectively. Participants in our study exhibited MPD changes of 0.8–1.0 mm from baseline. However, the V&K MRT test is unique to other tests and to the S&M MRT as between 3 and 5 mental rotations are made and held in memory when responding to a single trial. Previous work has linked changes in pupil dilation to working memory^51^ as the Locus Coeruleus (LC), which controls pupil dilation, is directly engaged in memory retrieval^52^. That the MRT in this study taxes memory processes to a larger degree when compared to other cognitive tasks might explain why participants are exhibiting larger pupil dilations, however further work examining changes in cognitive activity during the V&K MRT either directly (EEG) or indirectly (fNIRS) would corroborate such a claim.

Moreover, Aston-Jones and Cohen^53^ describe phasic and tonic modes of LC activity corresponding to different patterns in behaviour. Where the better understood phasic mode corresponds with task-relevant stimuli and is more tightly linked to focused attention or exploitation, tonic changes in pupil diameter have been investigated less and are more linked to a diffuse or exploration mode^36^. In the current study, participants are exploring between all 4 test images and referencing back to the standard, as opposed to the S&M MRT, where mental rotation comparisons are isolated and discrete. This taxing of cognition by the V&K MRT in this study may elicit pupil changes linked to a tonic mode of LC activity, where pupil diameter is seen to be larger and less varied across a task (Fig. 5A). This sustained processing yields an increase in the tonic activity^54^ and with higher task difficulty, performance can gradually degrade as subjects show higher distractibility with concomitantly large increases in pupil size observed^34,37,55–58^.

### Limitations

Our cohort of sports science students averaged 10.270 and 7.069 out of 20 on mirror and structural foil trials respectively. The relatively low average score of 17.353/40 by the participants in the current study may have resulted from a lack of practice on structural foil trials, a lack of experience with spatial and specifically, mental rotation tasks, and the method of scoring used.

It has been proposed that the structural difference between the standard and structural foils facilitates participants when solving these trials as they do not necessarily have to perform any mental rotation to differentiate between same and foil images^9^. As a result, participants are hypothesized to perform faster and more accurately on these trials compared to trials with mirror foils^48,59^. In the current study, participants did take longer to complete mirror compared to structural foil trials. However, we found contrary to previous reports that performance was superior for trials with mirror foils compared to trials with structural foils. We note that in the instructions presented to participants, which were adopted from Strong^13^, the existence of structural foil trials was not mentioned and the three sample problems provided were all mirror foil trials. This limitation may have led to superior scoring on mirror foil trials because participants had practiced only this style of foil. As no analyses between structural and mirror foil trials were provided by Strong, further investigation into the role of prior instructions on MRT performance would benefit the research area.

Previous work has demonstrated that individual scores on the MRT can differ depending on experience with tasks that require spatial cognitive abilities. For example, when scored such that points are awarded only when both answers on a given trial are correct, average MRT scores for individuals in areas of study not typically requiring a high degree of spatial ability range between 5/20^44^ and 10–12/24^14,48^. However, individuals from areas of organic chemistry, architecture, and graphics design score much higher, with scores ranging from approximately 11–20/24^8,12^ to 24–37/40^13,60^. The performance by individuals in the current study may have resulted from their limited experience with tasks requiring significant spatial cognitive ability. Future research may look to investigate the differences in gaze strategy, cognitive effort and performance among participants who vary in their experience with spatial cognitive tasks.

Finally, where many researchers score the V&K MRT with the criteria that points are awarded only for trials where both answers are correct or one answer is attempted and correct^8,11^, we were unable to identically score the computerized test in this study. On the computerized test, participants had to input 2 responses for each trial prior to moving onto the next trial and as a result, were not given the option to input one response *only*. As a result, it is impossible to determine for certain which trials participants were sure of one response and guessed on the other. Given that previous literature has suggested men are more likely to guess than women during mental rotation tasks^61^ capturing scores where one trial is attempted correctly might aid in differentiating performance between sexes.

## Conclusion

Overall, this study provides further evidence that the nature of sex differences on the V&K MRT is complex, with a number of factors potentially involved beyond mental rotation ability. We show for the first time, that participants may be adopting a leaping strategy including the strategy as evidenced by their differential fixation patterns on mirror and structural foil trials. Finally, this study showed that unlike the S&M MRT, pupillometry measures indicate that the V&K MRT may be exceedingly difficult for participants of both sexes who do not have an educational background associated with superior spatial ability. Moreover, the original and V&K MRTs may differentially recruit phasic and tonic modes of Locus Coeruleus activity, however further research specifically examining activity in this area will be required to confirm this. This research highlights the added value of eye tracking to illuminate the strategies employed during cognitive tasks and expands the debate over the nature of sex differences on mental rotation tasks. Moreover, it suggests that gaze and pupillometry measures may help to elucidate whether different strategies during cognitive tests might be adopted by individuals with cognitive impairments and as a result, may be used as a biological diagnostic marker of cognitive impairment.

## Methods

### Participants

One hundred (47 Male) young healthy undergraduate and postgraduate level psychology and sports science students from the University of Limerick (UL) volunteered to participate in the study. Participants were recruited from classes at UL to ensure a common academic background between males and females. All recruited participants were young healthy adults, between 18 and 40 years of age and each provided informed written consent prior to participation in the study. Participants were excluded from participating if they indicated they suffered from any neurological or neuromuscular disorder. All study procedures were approved by the local ethics committee of the Faculty of Education and Health Sciences (EHSREC) at the University of Limerick, Ireland in accordance with the Declaration of Helsinki.

### Computerized vandenburg mental rotations test (MRT)

Each participant completed a previously validated 20 trial computerized MRT test^13^. For 10 of the trials, two of the test images were mirror foils of the standard image which could not be rotated into congruency and for the other ten trials, two of the images were structural foils, with an end tail oriented in a different direction to the standard image. Trials were presented such that two mirror foil trials were followed by two structural foil trials and vice versa. For each of the twenty trials, participants were required to identify which two of the four test images were identical rotated versions of a standard image (see Fig. 1 for two sample trials)

### Protocol

Each participant began by completing the Edinburgh handedness questionnaire^62^. Then, they were positioned in front of a computer screen and presented instructions for how to complete trials on the MRT test, including three sample trials for practice. Participants were instructed to read and ensure they understood the instructions and to complete the sample trials. They were able to ask trials about the protocol during this time.

Following the instruction and practice phase, each participant’s gaze was calibrated to the computer screen using a Tobii X3-120 eye tracker and Tobii Studio (*v3*.*4*.*8*, Danderyd, Sweden) software. A 5-point calibration was used for gaze calibration and participants rested their head in a chin rest mounted to the table approximately 60 cm away from the computer screen (24 inch monitor). The chin rest was utilised throughout the testing phase of the experiment to minimize head movements and improve gaze recordings. All participants performed the test at the same computer station, in the same lab, and under the same monitor settings. Luminance was controlled and measured to be 90.0 ± 0.0 lux across all trials on the MRT using a Testoterm^TM^ 0500 Lux-Meter.

After completing eye tracking calibration, participants fixated on a small black crosshair in the middle of the screen prior to reading the task instructions (See Supplementary File). Time to complete the test was calculated from the final answer for trial 20 or until 15 minutes elapsed from the time of the SPACE key press to initiate the test. Gaze and keypress event data were recorded at 120 Hz and pupillometry data were recorded at 40 Hz.

### Data processing

#### Handedness

Responses from each participant’s handedness questionnaire were tallied and scored to provide a laterality quotient (LQ). Each participant’s handedness was then determined based on their LQ:

Right Handed: LQ > 40

Ambidextrous: −40 < LQ < 40

Left Handed: LQ < −40

Due to the low number of left handed (n = 10) and ambidextrous (n = 7) individuals and the importance of controlling analyses to include only right handed individuals^63–65^, left handed and ambidextrous participants were excluded from further analyses. One further right handed individual who reported they suffered from a neurological disorder was also excluded.

### MRT performance

Each participant’s responses were tallied and a total score (Score: All Correct) was calculated for the entire test (max score: 40), the 10 mirror foil trials (max score: 20) and the 10 structural trials (max score: 20). A secondary score (Score: 2 Correct) was also computed in an attempt to control for guessing^11^ whereby participants scored a single point only when both answers for a given trial were correct (max score total = 20; max score mirror foils = 10; max score structural foils = 10). A participant’s data were excluded from further analyses when they responded with keys other than F1, F2, F3 and F4 (10 participants). The time to complete every trial was recorded and from this, a time to completion (TTC) was calculated, in minutes, by summating trial completions times for the entire test, the mirror foil trials and the structural foil trials of the test.

### Eye tracking

#### Fixation data

Gaze coordinates for the left and right eyes were averaged and applied to the I-VT (Velocity-Threshold identification) fixation filter within Tobii Studio software. The I-VT fixation filter classifies fixations according to the velocity of directional shifts of the eyes in visual °/s. First, gaps in the data recording less than 75 ms were linearly interpolated within Tobii Studio according to Komogortsev and colleagues^65^, who determined this to be the minimum blink duration. Data were then classified by the I-VT filter in Tobii Studio to a fixation event when the velocity between adjacent gaze positions was less than 30°/s, in accordance with previous research^65–67^. When the velocity between data samples exceeded 30°/s, these data were classified under saccade events.

Following the classification of fixations within Tobii, areas of interest (AOIs) were created over the images presented in each trial. The AOIs were grouped based on whether they represented the standard image in mirror foil trials, the standard image in structural foil trials, the two images that were identical to the standard (same) for mirror foil trials, the same images for structural foil trials, the two mirror foil responses in each mirror foil trial or the two structural foil responses in each structural foil trial. Tobii Studio was then used to calculate, for each participant, the total fixation duration (TFD) and the total fixation count (FC) within each AOI group. Finally, in order to determine whether participants were directing their gaze more toward same versus foil test images, we calculated a Same:Foil ratio whereby the TFD or FC on Same AOIs was divided by the respective TFD or FC on Foil AOIs. In this way, a ratio larger than 1 suggested a participant spent more time fixated on, or directed a larger number of fixations toward, images that were identical rotated versions of the standard image (correct responses), whereas a ratio less than 1 suggested more time fixated on, or a larger number of fixations toward, the foil images of the standard (incorrect responses).

#### Pupillometry data

Pupil diameter data at each time frame were averaged between the left and right eyes. Where recordings were not available for both eyes the pupil diameter for a single eye was used, the pupil diameter of the right and left eye being highly correlated^68^. Following Lemercier and colleague^69^, a 10-point average filter was used to filter the data and a cubic spline was used to interpolate missing data over a maximum gap of 10 frames in Visual 3D software^23^. To represent changes in pupil diameter relative to a baseline for each participant, pupil diameter data were aligned relative to the average pupil dilation during the final 2 seconds of the visual baseline period, when participants fixated on the crosshairs. Where a participant had no pupil data recorded during the baseline period, their data were excluded from further analyses (2 participants). Then, the max pupil diameter (ΔMPD) was extracted from the window of time it took a participant to answer each of the 20 trials.

### Data analyses

Following exclusions, data from 70 participants (Male: N = 32; Age = 23.1 ± 4.29 years, Mean ± SD)(Female: N = 38; Age = 23.0 ± 4.93 years, Mean ± SD) were analysed (see Table 1). Males and females did not significantly differ in age (t(68) = 0.080, p = 0.937). Mixed-effects ANOVA with a between-subjects factor of *Sex* (male/female) and within-subjects factors of *Foil Type* (Mirror and Structural) was used to determine whether differences existed within each dependent variable. Greenhouse-Geisser corrections were used when Mauchly’s Test for Sphericity was significant and effect sizes are given by generalized eta squared (*η*^2^_*G*_)^70^. Data are represented as means ± 95% CI and can be found in Table 1.

## Supplementary information

Task Instructions for completing the V&K MRT

## Data Availability

The datasets generated during and/or analysed during the current study are available from the corresponding author on reasonable request.
