# Supplementary material for: Investigating sex differences, cognitive effort, strategy, and performance on a computerised version of the mental rotations test via eye tracking
Source: Sci Rep. 2019 Dec 19;9:19430. doi: 10.1038/s41598-019-56041-6 (PMC6923419; doi:10.1038/s41598-019-56041-6)
Supplement: Supplementary file 1 — Task Instructions for completing the V&K MRT [file 41598_2019_56041_MOESM1_ESM.docx]

**Investigating sex differences, cognitive effort, strategy, and performance on a computerised version of the mental rotations test via eye tracking**

*Adam J. Toth^1,2^, Mark J. Campbell*^1,2^*

^1^*Department of Physical Education & Sport Sciences, University of Limerick, Castletroy, Limerick, IRELAND*

^2^*Lero, Irish Software Research Centre, University of Limerick, Castletroy, Limerick, IRELAND*

*Corresponding Author

Dr. Mark J. Campbell

Department of Physical Education and Sport Sciences

Room p1041 PESS Building

University of Limerick, Limerick, Ireland

Phone: +353 61 234944

E-mail: [mark.campbell@ul.ie](mailto:mark.campbell@ul.ie)

**Task Instructions for completing the V&K MRT**

After completing eye tracking calibration, participants fixated on a small black crosshair in the middle of the screen prior to reading the following instructions:

*‘You are about to begin the mental Rotations Test (MRT).*

*There are 20 Questions.*

*As During practice, please indicate which TWO of the four test images are the SAME rotated versions of the standard image. Indicate your responses by pressing two of F1, F2, F3, and F4.*

*You have a MAXIMUM of 15 MINUTES to complete the test.*

*When you are ready to begin, please press the SPACE button on the keyboard.’*
